# 3D ultrasound guidance for radiofrequency ablation in an anthropomorphic thyroid nodule phantom

**DOI:** 10.1186/s41747-024-00513-6

**Published:** 2024-10-14

**Authors:** Tim Boers, Sicco J. Braak, Wyger M. Brink, Michel Versluis, Srirang Manohar

**Affiliations:** 1 Multi-Modality Medical Imaging Group, TechMed Centre, University of Twente, Enschede, The Netherlands; 2 Department of Radiology, Ziekenhuisgroep Twente, Almelo, The Netherlands; 3 Magnetic Detection and Imaging group, TechMed Centre, University of Twente, Enschede, The Netherlands; 4 Physics of Fluids group, TechMed Centre, University of Twente, Enschede, The Netherlands

**Keywords:** Imaging (three-dimensional), Phantoms (imaging), Radiofrequency ablation, Thyroid nodule, Ultrasonography (interventional)

## Abstract

**Background:**

The use of two-dimensional (2D) ultrasound for guiding radiofrequency ablation (RFA) of benign thyroid nodules presents limitations, including the inability to monitor the entire treatment volume and operator dependency in electrode positioning. We compared three-dimensional (3D)-guided RFA using a matrix ultrasound transducer with conventional 2D-ultrasound guidance in an anthropomorphic thyroid nodule phantom incorporated additionally with temperature-sensitive albumin.

**Methods:**

Twenty-four phantoms with 48 nodules were constructed and ablated by an experienced radiologist using either 2D- or 3D-ultrasound guidance. Postablation T2-weighted magnetic resonance imaging scans were acquired to determine the final ablation temperature distribution in the phantoms. These were used to analyze ablation parameters, such as the nodule ablation percentage. Further, additional procedure parameters, such as dominant/non-dominant hand use, were recorded.

**Results:**

Nonsignificant trends towards lower ablated volumes for both within (74.4 ± 9.1% (median ± interquartile range) *versus* 78.8 ± 11.8%) and outside of the nodule (0.35 ± 0.18 mL *versus* 0.45 ± 0.46 mL), along with lower variances in performance, were noted for the 3D-guided ablation. For the total ablation percentage, 2D-guided dominant hand ablation performed better than 2D-guided non-dominant hand ablation (81.0% *versus* 73.2%, *p* = 0.045), while there was no significant effect in the hand comparison for 3D-guided ablation.

**Conclusion:**

3D-ultrasound-guided RFA showed no significantly different results compared to 2D guidance, while 3D ultrasound showed a reduced variance in RFA. A significant reduction in operator-ablating hand dependence was observed when using 3D guidance. Further research into the use of 3D ultrasound for RFA is warranted.

**Relevance statement:**

Using 3D ultrasound for thyroid nodule RFA could improve the clinical outcome. A platform that creates 3D data could be used for thyroid diagnosis, therapy planning, and navigational tools.

**Key Points:**

- Twenty-four in-house-developed thyroid nodule phantoms with 48 nodules were constructed.
- RFA was performed under 2D- or 3D-ultrasound guidance.
- 3D- and 2D ultrasound-guided RFAs showed comparable performance.
- Real-time dual-plane imaging may offer an improved overview of the ablation zone and aid electrode positioning.
- Dominant and non-dominant hand 3D-ultrasound-guided RFA outcomes were comparable.

**Graphical Abstract:**

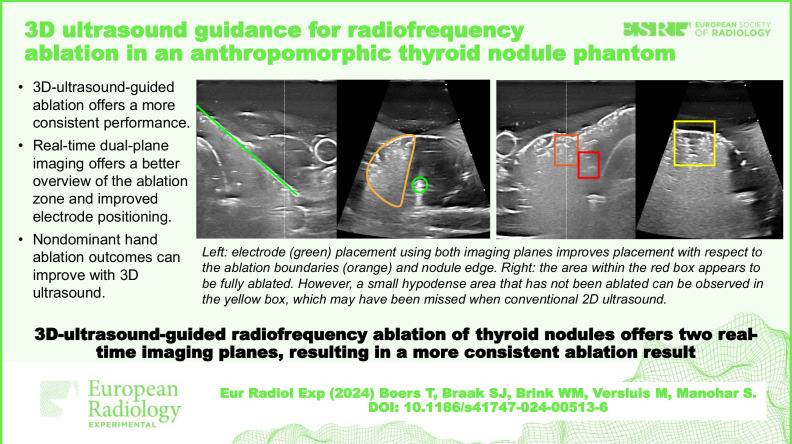

## Background

Thyroid nodule management typically utilizes two-dimensional (2D) ultrasound imaging during diagnosis, treatment, and follow-up [1]. As the thyroid is a relatively superficial structure, ultrasound offers a high-resolution view, allowing for initial nodule differentiation. Additionally, the real-time capacity of ultrasound enables image guidance for needle-based interventions such as fine needle aspirations and radiofrequency ablation (RFA). However, 2D ultrasound for thyroid nodules is limited by its measurement variability of up to 30% during thyroid and nodule assessment [2, 3], and it has a limited field of view during interventions. Furthermore, it restricts the possibilities for analysis improvement to only the 2D planar domain, impacting the accuracy of the initial diagnosis and, thus, treatment optimization, also including RFA, where the limited field of view makes it difficult to accurately position the electrode and makes it practically impossible to continuously monitor the entire treatment volume. Lastly, during follow-up, it is difficult to directly compare ablated and unablated regions over time, as these regions are highly irregular [4] and manual probe positioning is not sufficiently consistent [3, 5].

Real-time three-dimensional (3D) ultrasound may solve these issues by offering volumetric imaging, which is inherently less dependent on probe positioning. The use of 3D ultrasound in thyroid nodule management has been studied but its adoption in clinical practice and guidelines is still very limited [1]. Studies have shown the advantages of 3D ultrasound in determining thyroid and nodule volumes [6, 7], indicating that it is as accurate as low-dose computed tomography [8]. When compared to conventional 2D ultrasound, 3D ultrasound reduces observer variability [9–11] and improves sensitivity in detecting malignancies [11]. These studies were all conducted using mechanically swept linear transducers, whereas nowadays matrix transducers are available that utilize a large array of elements to produce a 3D ultrasound image, improving spatial resolution when compared to mechanically swept linear transducers, which results in better volume estimations [12]. In addition, matrix transducers offer an increase in frame rate, *i.e*., the number of volumes per second that can be produced, a relevant technical issue when 3D ultrasound is applied to diagnostic or therapeutic interventions such as fine needle aspiration or RFA [12]. 3D-ultrasound guidance has been studied in liver and prostate ablation studies as a fusion modality or stand-alone, where it has shown promise in treatment planning and interventional navigation [13–17].

Moreover, 3D ultrasound may impact the ablation results of the non-dominant hand *versus* the dominant hand: as for 2D ultrasound-guided ablation, these ablation results may possibly differ [18]. This is clinically relevant if we consider that thyroid nodules can appear in both lobes and isthmus and that the operator is positioned at the patient’s head; both hands of the operator must be adept in ablation. When the ablation results of the non-dominant hand are worse than those of the dominant hand, additional training (or dedicated tools) may be required for improvement.

In this work, we compare a linear transducer (2D ultrasound) with a matrix transducer (3D ultrasound) for image-guided RFA in an in-house-developed anthropomorphic thyroid nodule phantom [19], allowing for an accurate and non-destructive evaluation of the ablation procedure. To our knowledge, this is the first work to systematically compare 2D with 3D ultrasound-guided RFA in the context of thyroid nodules, performed in a carefully controlled “in-phantom” laboratory setting.

## Methods

**Fig. 1:**
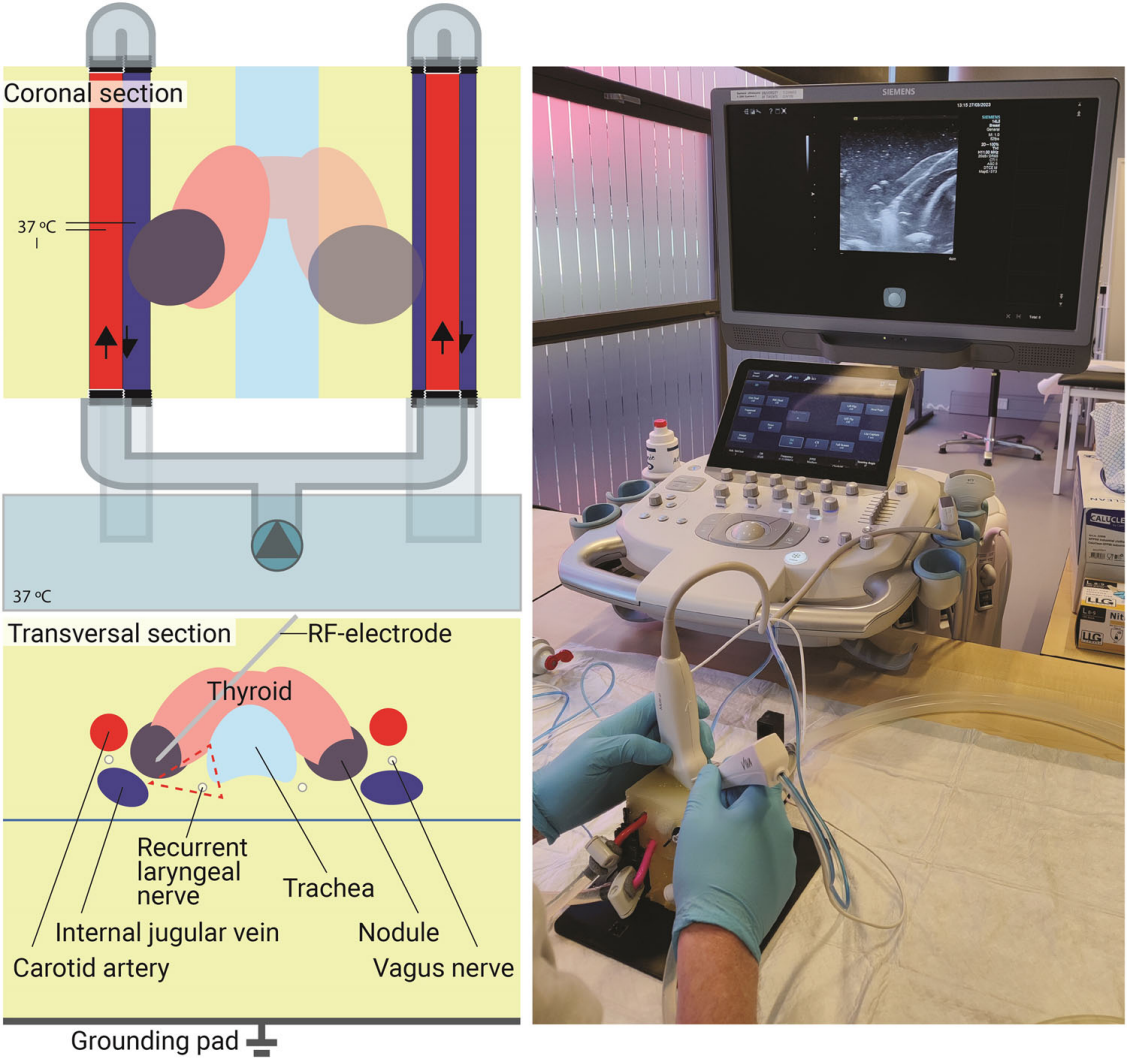
Setup used for the radiofrequency ablation procedure. Adapted with permission from the publishers of *Medical Physics* [19]. The coronal and transversal sections show a schematic drawing of the phantom, ablation, and flow setup, with the anthropomorphic phantom and supporting gel block (light yellow), the water bath, continuous flow pump, and silicone tubing (transparent blue), and the danger triangle (area indicated by the dashed red line). The right photograph shows the preablation setup, showing the phantom and supporting gel block on the bottom, with the pink thin-walled vessels running through it, and the ablation electrode and linear ultrasound transducer on top

For this study, we used an anthropomorphic thyroid nodule phantom developed by Boers et al [19], where the design, production, and characterization processes are detailed. Briefly, the phantom consists of polyacrylamide gel mixed with a temperature-sensitive albumin built up by a casting process using 3D-printed molds. The thyroid mold is based on the segmentation of a physiological human volunteer magnetic resonance imaging (MRI) scan. The nodules were digitally sculpted and merged with the thyroid model, one nodule in the left and one in the right lobe, in a caudal or lateral position. The phantom houses thin-walled tubes representing blood vessels that are connected with silicone tubing to a continuous flow pump, with which a water flow rate of 240 mL/min was created. The pump was placed in a temperature-controlled water bath (Anova Precision Pro cooker, Anova Applied Electronics, Inc., CA, USA) set at a temperature of 37 °C. The phantoms were pre-heated in this water bath to 37 °C prior to the ablation procedure. The RFA setup is illustrated in Fig. 1. The setup uses a platform to stabilize the phantom, and a gel block is positioned underneath to maintain an appropriate distance from the grounding pad. The supporting block is made from the same materials as the phantom.

For this study, 24 phantoms each containing two nodules were produced, with three nodule position variations: both nodules in a caudal position in the lobes, in a lateral and caudal position, or in a caudal and lateral position. Both nodules of the first 12 phantoms (4 for each position variation) were ablated with image guidance using a standard 14L5 linear transducer set to a working frequency of 11 MHz and interfaced to the Acuson S2000 ultrasound system (Siemens Healthineers, Erlangen, Germany). Both nodules of the last 12 phantoms were ablated with image guidance using an xL14-3 matrix ultrasound transducer interfaced with the EPIQ Elite ultrasound system (Philips Healthcare, Amsterdam, the Netherlands). The matrix transducer was used in dual-plane imaging mode, showing the transversal and sagittal planes side-by-side in real-time. Some use case images and a video of dual-plane imaging are shown for illustration (see the Supplementary Material for the video). It shows the insertion of the electrode into the thyroid phantom via the isthmus. The ablation procedures were performed by an experienced radiologist (S.B.), with over a hundred ablations performed clinically. To evaluate the effect of the dominant and non-dominant hands on the RFA result, each phantom had one nodule ablated using the dominant hand and the other nodule by the non-dominant hand.

For the RFA, two monopolar electrodes (18 G, 5–20 mm set at 10 mm active zone length, 360° active zone, internally liquid-cooled with a sharp electrode tip, operating at 480 kHz) were used in combination with the VIVA RF-system (both from STARmed Co., Ltd., Goyang, South Korea). The electrodes were re-used and thoroughly cleaned in between ablations to prevent conductivity impairment. The gel block and phantom were placed on a single grounding pad. The generator power was set at 50 W. RFA was performed following the clinical guidelines: a transisthmic approach and the multiple-overlapping-shots technique were used [20]. Furthermore, the danger triangle was avoided, which is known to potentially lead to damage to the recurrent laryngeal nerve. The ablation was considered complete for one position when the ohmic resistance of the electrode was at least over 150 ohms. The procedure was continued until the nodule was “fully” ablated, based on the hyperechoic appearance of gas bubbles in the entire nodule, with the exception of the danger triangle. The ablation percentage, volume ablated outside the nodule, count of critical structures hit, total time taken for ablation (defined as the moment from electrode insertion to electrode removal), dominant or non-dominant hand ablation, as well as the total amount of kcal used, were recorded during the procedure.

The ablation performance was evaluated by performing MRI-based T2 mapping of the phantoms. A 1.5-T system was used (Aera, Siemens Healthineers, Erlangen, Germany) equipped with a 16-channel head coil array for signal reception. A series of 3D T2-weighted fast-spin-echo sequences were chosen with increments in the echo time to encode T2. The following settings were used: field of view 192 × 192 × 64 mm^3^; isotropic voxel size 1 mm^3^; repetition time 1,000 ms; echo time 80–400 ms in steps of 80 ms; number of refocusing pulses 64; echo spacing 10 ms. The vessel structures were oriented parallel to the direction of the main magnetic field (*B*_0_) to prevent susceptibility artifacts from overlapping with the internal structures of the phantom. The data were fitted to a monoexponential decay model in a voxel-wise manner using MATLAB^®^ (R2022b, Mathworks, Natick, MA, USA) to obtain quantitative T2 maps.

The quantitative T2 maps were converted into temperature maps using reference data acquired from reference spherical phantoms [19]. The resulting temperature-to-T2 data were then analyzed using a fifth-order polynomial fitting procedure for temperatures between 35 and 90 °C, defined specifically for this phantom recipe and Magnetic Resonance Imaging sequence [19]. Thereafter, the ablation zone was segmented at a threshold level of 50 °C. The MATLAB^®^ function imfill was used to fill up cavities that were created by the electrode. The 50 °C maps were loaded into 3D Slicer (Slicer version 5.2, www.slicer.org) [21], where the corresponding 3D models were visually matched. MATLAB^®^ (R2022b, Mathworks, Natick, MA, USA) was used to calculate the overlay percentage between both models (nodule ablation percentage) and to subtract the nodule model from the ablation segmentation model (volume ablated outside the nodule). To show where overablation occurred, the 3D rendering of the ablated areas that were visually matched to their corresponding preablation 3D models of the nodules was combined with the three scan orientations based on the ultrasound volume data.

All data is presented with medians and interquartile ranges based on Shapiro-Wilk tests for normality. A Mann–Whitney *U*-test was performed to determine significance (threshold set at alpha = 0.05). SPSS (IBM Corp., version 28.0, released in 2021, Armonk, NY, United States) was used for the calculation of the statistical tests. To assess whether there is a learning curve, the case numbers of the dominant and non-dominant hand ablations were correlated to the 2D and 3D-guided nodule ablation percentages, the amount of energy used, and the volume ablated outside of the nodule. For this assessment, coefficients of determination and Spearman’s *ρ* correlation coefficients were determined.

## Results

**Fig. 2:**
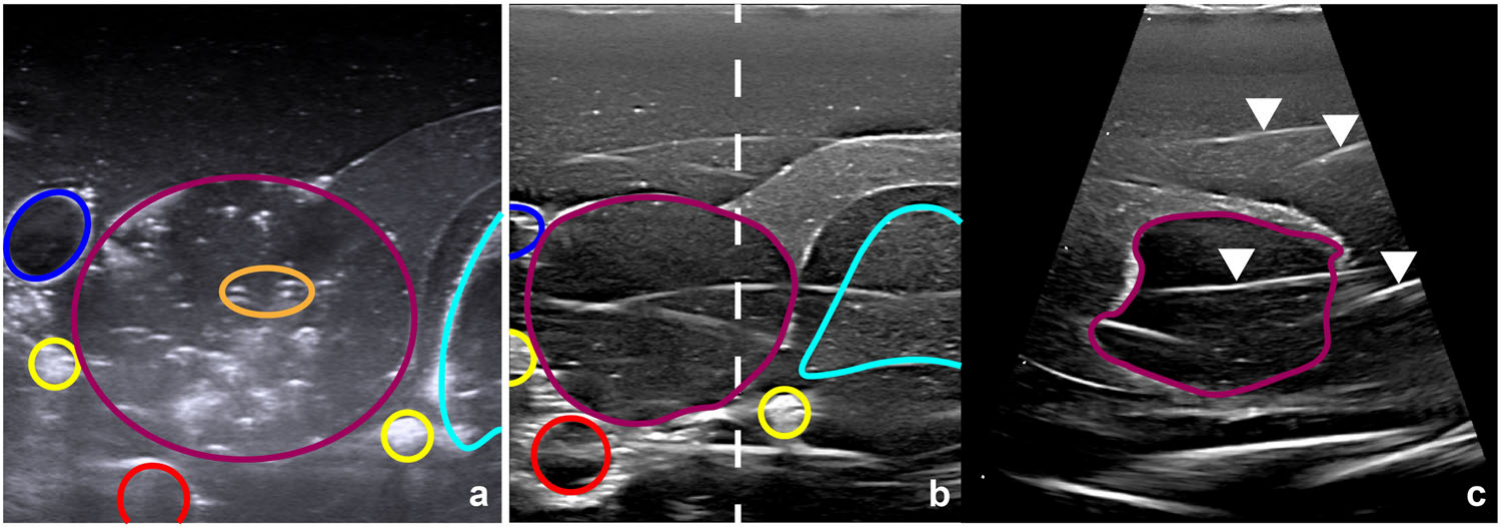
**a** Conventional two-dimensional ultrasound image. **b**, **c** Three-dimensional matrix transducer dual-plane images. The white dashed line shows the acquisition position of the second plane. The conventional and matrix images show two different phantom nodules before ablation. The following structures are highlighted: internal jugular vein (dark blue), nerves (yellow), carotid artery (red), nodule (purple), trachea (light blue), bubble and silicium dioxide artefacts (orange) and streak artefacts (white arrows, right)

**Fig. 3:**
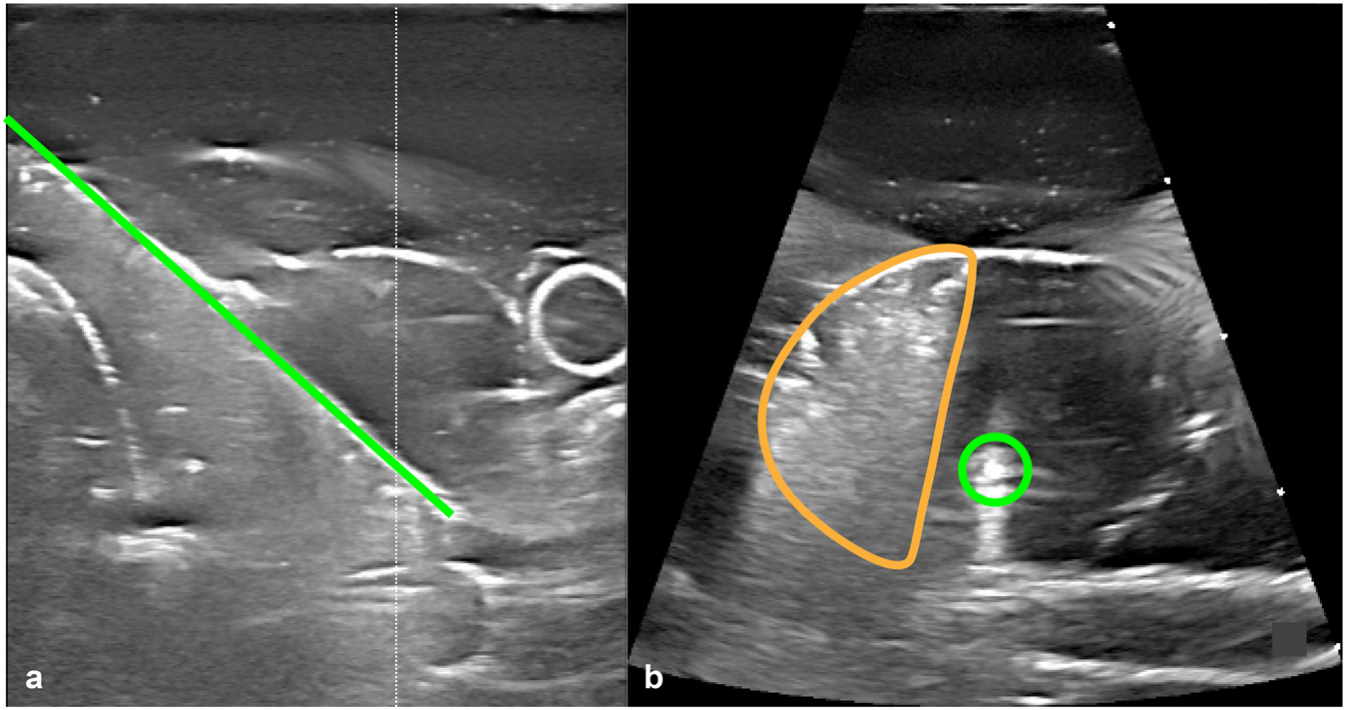
An example of the use of dual-plane imaging (three-dimensional ultrasound), the electrode (green) was placed using both planes (**a**, **b**) allowing for easy placement with respect to the ablation boundaries (orange) and nodule edge

**Fig. 4:**
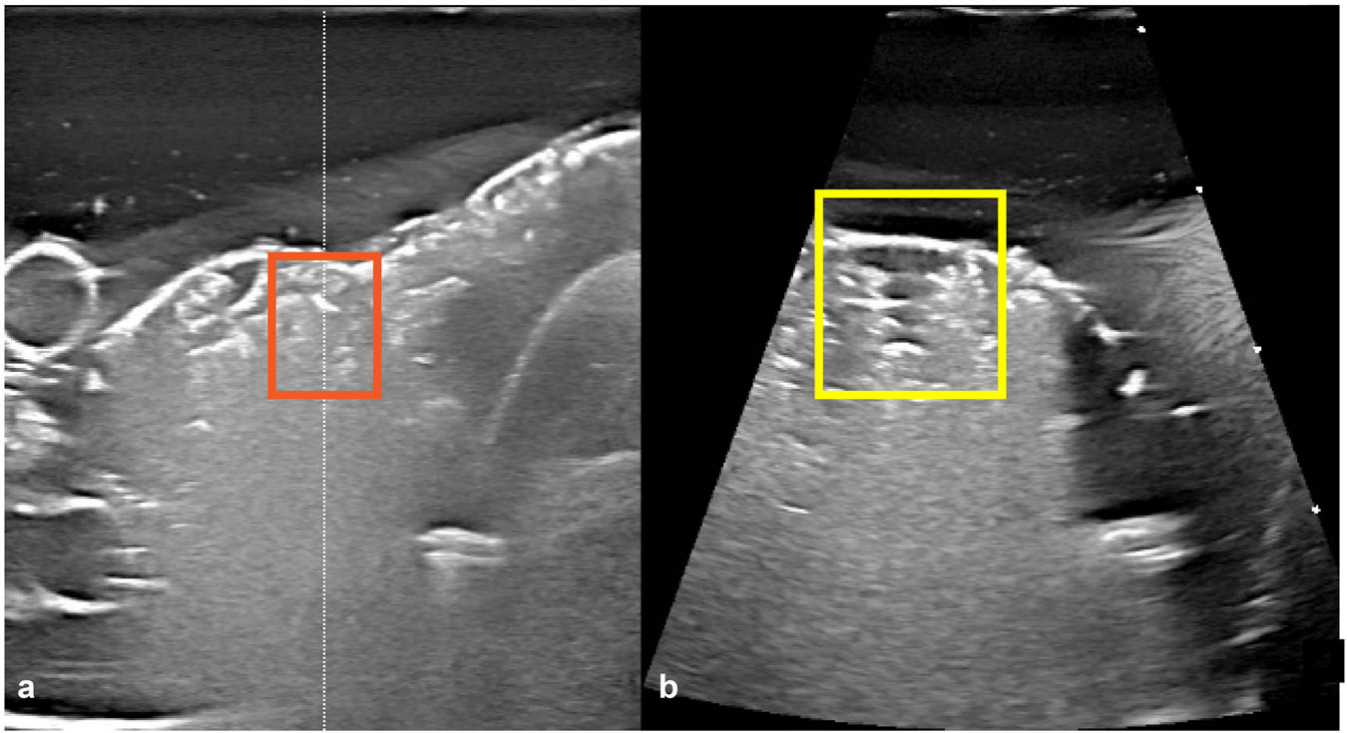
An example of the dual imaging plane potential (three-dimensional ultrasound), where on the standard transversal plane (**a**), the area surrounding the white dashed line (orange box) appears to be fully ablated. However, the sagittal plane (**b**) shows a small hypodense area (yellow square) that has not been ablated

**Fig. 5:**
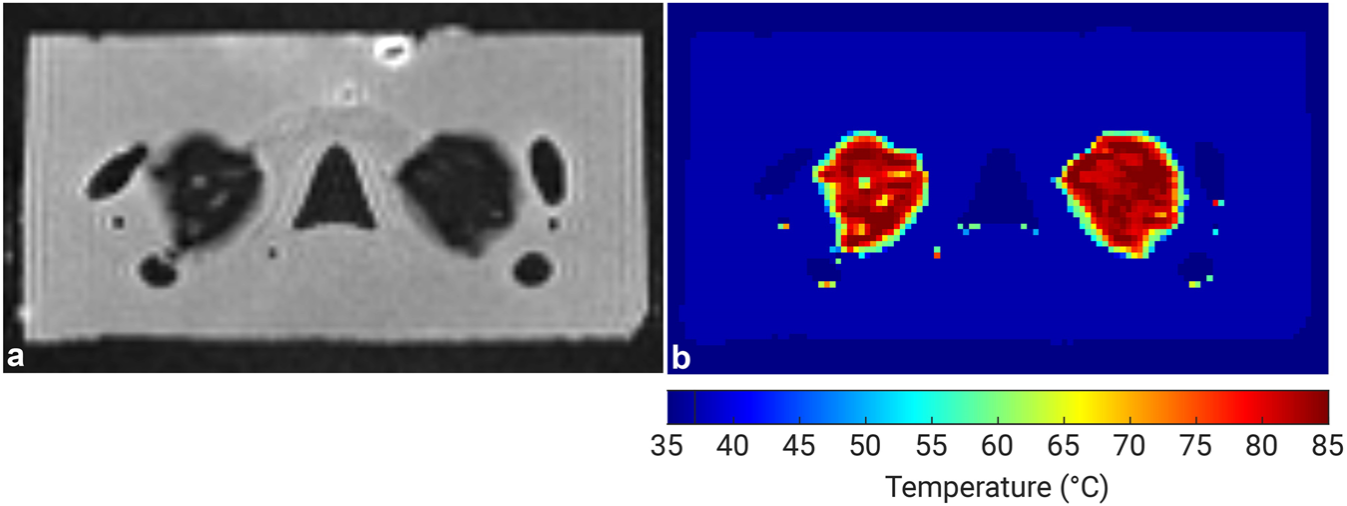
**a** Postablation T_2_-weighted magnetic resonance imaging scan (echo time = 400 ms) showing the lack of signal in the ablated areas. **b** The corresponding temperature map of the ablated areas

**Fig. 6:**
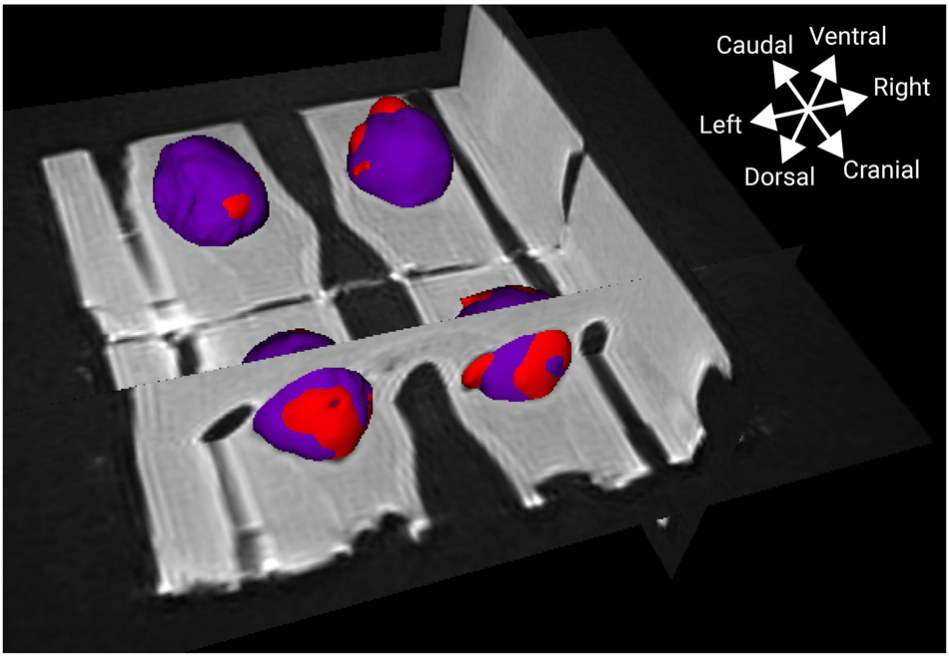
A combination of the digital three-dimensional model of the nodule (purple) with the corresponding rendered segmented ablated areas (red). Where red is visible, it indicates ablation outside the nodule

A total of 48 nodules were ablated, half with 2D ultrasound guidance and half with the 3D matrix transducer. Figure 2 shows examples of ultrasound images obtained using these modalities. We note the clear delineations of the thyroid, nodule, blood vessels, and trachea for both transducers. In the 3D ultrasound images, streak artifacts are present. Figures 3 and 4 show two use cases for dual-plane imaging, one placing the electrode with respect to already ablated areas and one inspecting the ablation area for unablated parts. Figure 5 shows a Postablation T2-weighted MRI scan and the corresponding temperature maps evaluated from the MRI scans. Note the steep temperature drop-off at the edge of the ablation zone, which was also observed in earlier studies [12]. Figure 6 presents an example visualization of a combination of the rendered ablated area, together with the corresponding digital 3D model of the nodule and the three ultrasound scan orientations.

**Fig. 7:**
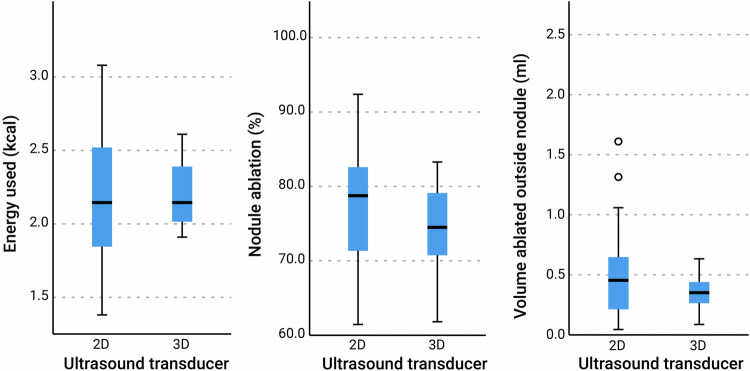
Boxplots showing the median and interquartile ranges for the two-dimensional *versus* three-dimensional ultrasound-guided radiofrequency ablation

The results from the volumetric analyses are shown in Tables 1 and 2. Shapiro-Wilk tests showed non-normally distributed data for most parameters. No significant differences were found when comparing the medians of the parameters of 2D and 3D-guided RFA. While these results indeed do not differ significantly, trends can be observed. A lower ablation percentage was found for 3D-guided RFA, in combination with a lower volume ablated outside the nodule and a half-minute longer ablation time. When looking at the interquartile range of the measured parameters, 3D-guided RFA was shown to have a smaller range than 2D-guided RFA (see also Fig. 7). In line with keeping the danger triangle-free, none of the nodules were fully ablated.

**Table 1:** Summary of ablation parameters 2D-guided *versus* 3D-guided ultrasound

Parameters	Conventional 2D ultrasound	Matrix 3D ultrasound	*p*-value^a^
Percentage of nodule ablated	78.8 (11.80)	74.4 (9.05)	0.216
Volume ablated outside of nodule (mL)	0.45 (0.46)	0.35 (0.18)	0.303
Count of critical structures hit (out of 24)	0	0	–
Energy delivered (kcal)	2.15 (0.75)	2.15 (0.41)	0.869
Total ablation time (min)	6.5 (2.5)	7 (2.0)	0.247

Data are given as medians (interquartile range)

*2D* Two-dimensional, *3D* Three-dimensional

^a^ Mann–Whitney *U*-test

**Table 2:** Summary of non-dominant *versus* dominant hand differences and their statistical results

Parameters	Non-dominant hand		Dominant hand		*p*-value^a^	
	2D	3D	2D	3D	2D	3D
Percentage of nodule ablated	73.2 (21.8)	72.0 (20.9)	81.0 (13.7)	76.3 (8.15)	**0.045**	0.219
Volume ablated outside of nodule (mL)	0.37 (0.40)	0.33 (0.23)	0.45 (0.70)	0.42 (0.16)	0.478	0.378
Energy delivered (kcal)	2.01 (0.48)	2.06 (0.16)	2.42 (0.34)	2.30 (0.5)	**0.006**	0.060
Total ablation time (min)	6.0 (1.75)	6.5 (2.75)	7.0 (1.75)	7.0 (2.50)	**0.017**	0.319

Data are given as medians (interquartile range)

*2D* Two-dimensional transducer, *3D* Three-dimensional transduce

^a^ Mann–Whitney *U*-test (significant values in bold characters)

**Fig. 8:**
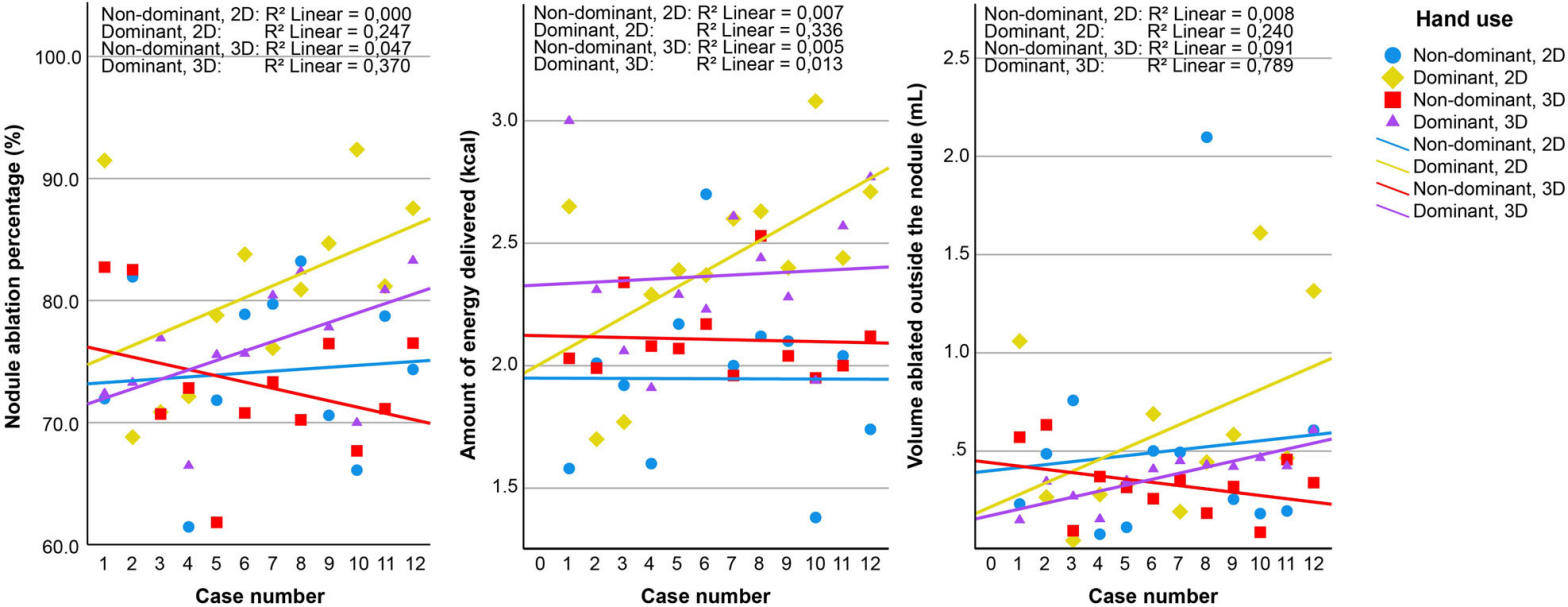
Linear fit lines for the dominant and non-dominant hand ablations. The nodule ablation percentage, amount of energy delivered, and the volume ablated outside of the nodule are plotted against the case numbers for both 2D- and 3D-guided ablations performed with the dominant and non-dominant hand. 2D, Two-dimensional; 3D Three-dimensional

When comparing the ablation performance of the dominant *versus* the non-dominant ablating hand in 2D-guided RFA, significant differences were found in favor of the dominant hand. The total ablation percentage, the number of kcal used, and the time taken for a complete ablation varied significantly, while it did not for the volume ablated outside the nodule (see Table 2). In contrast, the phantom results for the 3D-guided RFA demonstrate no significant differences in the ablation performance of the dominant *versus* non-dominant ablating hand (see also Table 2). In addition, the correlation analysis between the number of ablations performed and the ablation outcome parameters is shown in Table 3. In the 2D-guided ablations performed with the dominant hand, the number of cases performed showed a correlation only with the amount of ablation energy applied (*ρ* = 0.58, *p* = 0.048, *R*^2^ linear = 0.336), while it did not correlate with the nodule ablation percentage and the volume ablated outside the nodule. Conversely, in the 3D-guided ablations performed with the dominant hand, the number of cases performed showed a correlation both with the ablation percentage (*ρ* = 0.61, *p* = 0.036, *R*^2^ linear = 0.370) and the volume ablated outside the nodule (*ρ* = 0.89, *p* < 0.001, *R*^2^ linear = 0.789). However, it did not correlate with the amount of ablation energy applied. To illustrate these learning curves, in Fig. 8, the nodule ablation percentage is plotted against the case number, showing a steeper learning curve for the dominant hand when compared to the non-dominant hand for both 2D- and 3D-guided ablations.

**Table 3:** Learning curve assessment using the Spearman’s *ρ* correlation coefficient of the case numbers of the dominant and non-dominant hand 2D- and 3D-guided ablations with the treatment outcome parameters

Cases	Nodule ablation percentage		Energy delivered		Volume ablated outside of the nodule	
	*ρ*-value	*p*-value^a^	*ρ*-value	*p*-value^a^	*ρ*-value	*p*-value^a^
Non-dominant hand 2D	0.007	0.983	0.084	0.795	0.091	0.779
Dominant hand 2D	0.497	0.101	0.580	**0.048**	0.490	0.106
Non-dominant hand 3D	-0.217	0.499	-0.070	0.829	-0.301	0.342
Dominant hand 3D	0.608	**0.036**	0.112	0.729	0.888	**<** **0.001**

*2D* Two-dimensional guidance, *3D* Three-dimensional guidance

^a^ Spearman’s *ρ* test (significant values in bold characters)

## Discussion

A total of 48 nodules in 24 thyroid phantoms have been ablated to compare 2D *versus* 3D ultrasound guidance during RFA of thyroid nodules. No significant differences were found when comparing the 2D- and 3D-guided RFA results, although a trend towards a lower ablated volume outside the nodule with a lower variance was found for the 3D-guided RFA. A comparison between using the non-dominant *versus* dominant ablating hand per transducer type showed significant differences in 2D-guided RFA performance in favor of the dominant hand, while these differences were not significant for 3D-guided RFA. A learning curve was found for the amount of energy introduced with 2D-guided ablation and for the nodule ablation percentage and volume ablated outside the nodule with 3D-guided ablations, albeit, only for the dominant hand (Table 3).

The trend towards a lower median ablated volume outside of the nodule with lower variance, when using 3D ultrasound guidance (Table 1), can originate from the convenience of two real-time scanning planes throughout the ablation, allowing for improved electrode placement. However, the lack of experience in interpreting the additional information may have also led to a lower ablated volume on the inside of the nodule. As the nodule ablation percentage correlates with the reduction in volume of the nodule over time, and thus with symptom relief for the patient, a high nodule ablation percentage is important. Conversely, a low as possible volume ablated outside the nodule is important to reduce the risk of damaging healthy thyroid tissue as well as blood vessels and nerves surrounding the thyroid and nodule. Ideally, a high nodule ablation percentage and low ablated volume outside the nodule are achieved. Additional training using 3D-ultrasound guidance, in combination with a computer-aided interventional system, can be the next step.

Two use cases were shown to illustrate the presentation of dual-plane images for nodule ablation as well as how they can be used. Dual-plane imaging may be helpful when positioning the electrode with respect to the nodule boundaries and previously ablated areas (Fig. 3) and in finding unablated parts of the nodule (Fig. 4).

The availability of continuous information (*i.e*., real-time image guidance) may also have contributed to the more consistent performance (Table 1): the electrode placement is more consistently accurate when using 3D guidance. Previous reports on 2D *versus* 3D ultrasound guidance in prostate biopsies showed an improvement in cancer detection with more biopsy cores containing malignant tissue when using 3D guidance [22]. Another study found improved accuracy and robustness for renal punctures [23]. A liver tumor phantom study comparing 2D and 3D ultrasound-guided punctures found improved accuracy for both experts and novices [24].

Achieving consistent results when performing RFA procedures in the thyroid requires extensive training, with at least 60 and preferably 90 finished ablations [25]. Our operator (SB) has performed, including this work and several test sessions, a total of 48 ablations on phantoms with 3D ultrasound guidance and may, therefore, still be considered learning to perform 3D-guided ablations. The determined correlation coefficients support the learning curves only partly, as the coefficients show no evidence of a learning curve for the non-dominant hand. However, they do suggest a learning curve for the dominant hand in both 2D and 3D-guided ablations (Table 3 and Fig. 8). As Table 3 shows the correlation coefficients (*i.e*., *R*-value) and Fig. 8 shows the coefficients of determination (*i.e*., *R*^2^-value), the values in Fig. 8 are lower due to this squaring. For the significant correlation coefficients, the increase in case numbers of the dominant hand can only explain approximately 35% of the encountered variation in nodule ablation percentage and the amount of energy delivered, meaning that the majority of the variation is not due to a learning effect.

For the volume ablated outside the nodule by the 3D-guided dominant hand, 78.9% of the increase was explained by the increase in case numbers, this means that with the increase in the number of performed ablations, more ablation is performed near the edges of the nodule resulting in an increase in volume ablated outside the nodule, which can be interpreted as a learning effect. These results emphasize that learning to perform 3D-guided ablations was not yet finished. As both dominant hand 2D and 3D-guided ablations show significant correlation with the case numbers, this may suggest the occurrence of two interplaying learning curves: one for using the 3D ultrasound transducer, and one for ablating with the phantom. The results from Table 3 and Fig. 8 may also suggest that the interventionist required more training, with 3D ultrasound, as more outcome parameters correlated to the cases performed, and with a higher coefficient of determination than for 2D ultrasound (Fig. 8). These learning curves impede the extrapolation of the results to the clinic, as the results cannot be attributed to the difference between ultrasound guidance alone. Therefore, the current results might change when the learning curve is completed.

If 3D ultrasound guidance can support the non-dominant hand in accurately performing ablations, the significant correlations of the ablation parameters with the case numbers should become insignificant, and the correlations between the ultrasound guidance type per hand and the ablation parameters should become stronger for the 2D-guided ablations and less strong for the 3D-guided ablations. A fair comparison between both ultrasound-guided ablations is best made when the learning curve factor is no longer present, thus training with the phantom for at least 60 ablations [25] before research is recommended. In order to reduce this number, the phantom could benefit from further optimization to better mimic the ablation experience in patients, such as the inclusion of muscle groups and more accurate matching of the echogenic presentation of the various tissues.

Nevertheless, our results on 3D guidance did not indicate a larger risk for the “patient” (Table 1) and may even be safer when this learning curve is completed. Further improvements to 3D ultrasound guidance may include the use of a navigational system, as in the case of, *e.g*., prostate biopsy procedures [22]. This should also include more elaborate preoperative planning that considers the size of the RFA zone to prevent damage to nearby vital structures. As most computer-aided intervention systems use volume data for planning and navigation, while most thyroid nodule patients typically have scans made using conventional ultrasound, 3D ultrasound is well-suited to create that volume data. Furthermore, using the 3D visualizations of the ablated areas in combination with the preablation nodule models and the three ultrasound scan orientations (Fig. 6), shows where ablation outside the nodule has occurred. This visual insight may help in evaluating ablations as well as expedite the learning process during training.

A benefit of using phantoms for training is the possibility of comparing the dominant hand to the non-dominant hand. With the dominant hand, for 2D-guided RFA, the operator was able to achieve a higher ablation percentage, which was reflected in a higher number of kcal that were applied to the gel as well as requiring more time to perform the ablation compared with the non-dominant hand (Table 2). This can be attributed to an increased level of confidence in achieving good electrode positioning with the dominant hand. Thus, more areas have been ablated with the dominant hand, which also explains why the ablation time is longer for the dominant hand (Table 2). The differences disappear for the 3D-guided RFA data, indicating a benefit of using dual-plane visualization in counteracting the dominant *versus* non-dominant hand difference. However, increasing the number of compared nodules can affect the outcome of the number of kcal applied, as the *p*-value (0.060, Table 2) is borderline. In a study on the difference between hands in needle positioning for pediatric surgeons, no difference was found in the main goal of the task. However, the non-dominant hand was moving faster and less economically [26]. This may have also been the case in this study. In an ultrasound-guided needle placement study, the difference between hands was more apparent for ultrasound novices, showing quicker and more accurate needle placement for the dominant hand [18]. Our operator (S.B.) is an expert in performing needle-based ablation, and the difference between the dominant and non-dominant hands was not significant when using 3D ultrasound. Despite this, the use of dual-plane imaging is different from the current standard of one single plane and should be trained before use on patients.

### This study involves some other factors

Only one operator performed the experiments, limiting generalizability. Nevertheless, the results are in line with the literature [18, 26], confirming the potential difference between the dominant and non-dominant hand in needle-based interventions.

The power of the study was calculated by expecting a 10-percentage point ablation difference between 2D- and 3D-guided ablations, which required 42 nodules. To be on the safer side, we chose 48 nodules, 24 per transducer. Subsequently, this also means 24 nodules per hand. However, it leaves only 12 nodules ablated per hand per transducer, which may have been just short of achieving full statistical power. Nevertheless, some significant results were found.

The two ultrasound probes acquire, process, filter, and reconstruct images differently. The literature discusses the presence of artifacts unique to 3D ultrasound [27, 28]. This has most likely resulted in more artifacts being visible in our 3D images as compared to 2D images (see Fig. 2). Note that these imaging systems are not optimized for phantoms; therefore, the observation of artifacts here cannot be directly translated to the *in vivo* case, though they are expected based on literature [27, 28]. Nevertheless, the artifacts did not obstruct the identification of the nodule borders, nor did they impair the final ablation results.

Based on our study, we cannot yet draw definitive conclusions on the superiority of one imaging technology over the other, as the total nodule ablation percentage did not improve. Nevertheless, we have shown that the use of a matrix transducer for 3D ultrasound can reduce the variance encountered with 2D-guided ablation as well as reduce the dependency on the dominant hand for 2D-guided ablations. Both the in-plane insertion in the transversal view as well as the out-of-plane insertion in the sagittal view are simultaneously shown. This may improve electrode placement with respect to the border of the nodule and the ablated area. Further, the dual-plane functionality offers a larger field of view than obtained with just a single plane, which, according to our operator, allowed for obtaining more comprehensive feedback on the ablation process. Further research should focus on the matrix transducer’s ability to create 3D datasets that can serve as patient models on which image-guided interventional systems can be based. Moreover, this system allows for preoperative planning based on said patient models, which could be the next step in the management of RFA for thyroid nodules.

In conclusion, 3D ultrasound-guided RFA shows similar ablation performance as 2D ultrasound-guided RFA. However, 3D ultrasound offers additional imaging information, which might improve electrode placement with respect to the target area, which explains the smaller variance observed in the ablation results. A significant reduction in operator-ablating hand dependence was also observed in 3D-guided RFA. These results warrant further research into the use of 3D ultrasound for thermal ablations.

## Supplementary information

Supplementary Video 1

**Additional file 1: Supplementary Video 1.** A matrix transducer recording of an RF-electrode placement from the isthmus into the phantom nodule, showing two real-time scanning planes at the same time. The transversal scanning plane (left) is parallel to the RF-electrode and shows the in-plane insertion (as well as the trachea on the left and the carotid artery on the right), the sagittal plane (right) is orthogonal to the RF-electrode and shows the out-of-plane insertion

## Data Availability

The datasets used and/or analyzed during the current study are available from the corresponding author upon reasonable request.

## Abbreviations

2D: Two dimensional
3D: Three dimensional
MRI: Magnetic resonance imaging
RF: Radiofrequency
RFA: RF ablation
