# Supplementary material for: 3D ultrasound guidance for radiofrequency ablation in an anthropomorphic thyroid nodule phantom
Source: Eur Radiol Exp. 2024 Oct 14;8:115. doi: 10.1186/s41747-024-00513-6 (PMC11473505; doi:10.1186/s41747-024-00513-6)
Supplement: Supplementary file 2 — Additional file 1: Supplementary Video 1. A matrix transducer recording of an RF-electrode placement from the isthmus into the phantom nodule, showing two real-time scanning planes at the same time. The transversal scanning plane (left) is parallel to the RF-electrode and shows the in-plane insertion (as well as the trachea on the left and the carotid artery on the right), the sagittal plane (right) is orthogonal to the RF-electrode and shows the out-of-plane insertion [file 41747_2024_513_MOESM2_ESM.pdf]

3D ultrasound guidance for radiofrequency ablation in an anthropomorphic thyroid nodule phantom

ELECTRONIC SUPPLEMENTARY MATERIAL

Supplementary Video 1.

A matrix transducer recording of an RF-electrode placement from the isthmus into the phantom nodule, showing two real-time scanning planes at the same time. The transversal scanning plane (left) is parallel to the RF-electrode and shows the in-plane insertion (as well as the trachea on the left and the carotid artery on the right), the sagittal plane (right) is orthogonal to the RF-electrode and shows the out-of-plane insertion.
